# The altered sputum microbiome profile in patients with moderate and severe COPD exacerbations, compared to the healthy group in the Indian population

**DOI:** 10.12688/f1000research.132220.4

**Published:** 2023-10-27

**Authors:** Druti Hazra, Fayaz SM, Kiran Chawla, Vitali Sintchenko, Elena Martinez, Rahul Magazine, Nayana Siddalingaiah

**Affiliations:** 1 Department of Microbiology, Kasturba Medical College, Manipal Academy of Higher Education, Manipal, Karnataka, 576104, India; 2 Department of Biotechnology, Manipal Institute of Technology, Manipal Academy of Higher Education, Manipal, Karnataka, 576104, India; 3 Centre for Infectious Diseases and Microbiology-Public Health, Westmead Hospital, Westmead, New South Wale, 2145, Australia; 4 Sydney Institute for Infectious Diseases, The University of Sydney, Sydney, New South Wales, 2145, Australia; 5 Department of Respiratory Medicine, Kasturba Medical College, Manipal Academy of Higher Education, Manipal, Karnataka, 576104, India

**Keywords:** Microbiome, chronic obstructive pulmonary disease, respiratory pathology, 16S rRNA gene sequencing, microbial populations

## Abstract

**Background:** Microbial culture-independent sequencing techniques have advanced our understanding of host-microbiome interactions in health and disease. The purpose of this study was to explore the dysbiosis of airway microbiota in patients with moderate or severe chronic obstructive pulmonary disease (COPD) and compare them with healthy controls.

**Methods:** The COPD patients were investigated for disease severity based on airflow limitations and divided into moderate (50%≤FEV1<80% predicted) and severe groups (FEV1<50% predicted). Spontaneous sputum samples were collected and, the V3-V4 regions of the 16S rRNA coding gene were sequenced to examine the microbiome profile of COPD and healthy participants.

**Results:** A total of 45 sputum samples were collected from 17 severe COPD, 12 moderate COPD cases, and 16 healthy volunteers. The bacterial alpha diversity (Shannon and Simpson’s index) significantly decreased in the moderate and severe COPD groups, compared to healthy samples. A significantly higher proportion of Firmicutes and Actinobacteria were present in moderate COPD, and Proteobacteria numbers were comparatively increased in severe COPD. In healthy samples, Bacteroidetes and Fusobacteria were more abundant in comparison to both the COPD groups. Among the most commonly detected 20 bacterial genera, *Streptococcus* was predominant among the COPD sputum samples, whereas *Prevotella* was the top genus in healthy controls. Linear discriminant analysis (LDA>2) revealed that marker genera like *Streptococcus* and *Rothia* were abundant in moderate COPD. For severe COPD, the genera *Pseudomonas*and *Leptotrichia* were most prevalent, whereas *Fusobacterium* and *Prevotella* were dominant in the healthy group.

**Conclusions:** Our findings suggest a significant dysbiosis of the respiratory microbiome in COPD patients. The decreased microbial diversity may influence the host immune response and provide microbiological biomarkers for the diagnosis and monitoring of COPD.

## Introduction

Chronic obstructive pulmonary disease (COPD) is a heterogenous lung pathology, manifesting with persistent and progressive respiratory symptoms, airway obstruction, and inflammation due to structural abnormalities. ^1^ COPD is one of the leading causes of mortality and morbidity globally and its burden is predicted to rise further in the upcoming years due to constant exposure to air pollution, respiratory pathogens, and the growing elderly population. ^1^^–^^3^ COPD is a treatable but incurable disease, and often switches between a stable to an exacerbated state disease. ^4^ The frequent exacerbation and worsening of respiratory symptoms affect the individual quality of life with a significant socioeconomic burden and impose a huge healthcare management cost. ^5^^,^^6^ The progression of COPD often leads to chronic inflammation and major destruction of the lung and airway, which disrupts pulmonary microbiome homeostasis. Commensal microbes play a crucial role in innate immune regulation, protecting against invading pathogens and maintaining epithelial integrity. The recent advancement of culture-independent next-generation sequencing techniques have uncovered the diverse microbial communities colonizing the respiratory mucosa and recognized their roles in health and disease. ^7^^–^^9^

In the past, several studies have evaluated lung microbiome composition and its association with COPD manifestations. The changes in bacterial diversity and several significant taxa have been identified in COPD patients, which play a role in disease progression. A high heterogeneity has been observed among these studies, potentially attributed to the underlying health conditions of selected populations and geographical variations. However, the changes in the sputum microbial profile in the Indian population with COPD are not well understood. This study aimed to evaluate the changes in lung microbiome diversity of patients with moderate or severe COPD and compare them with the microbiomes of healthy controls.

## Methods

### Ethical considerations

This study was approved by the Kasturba Medical College and Kasturba Hospital Institutional Ethics Committee [IEC: 479/2019].

### Consent to participate

Written informed consent was obtained from all the participants.

### Study design and population

To conduct this prospective observational study, sputum samples were collected from eligible COPD participants, who presented to our hospital and were diagnosed with COPD in accordance with the 2019 Global Initiative for Chronic Obstructive Lung Disease (GOLD) guideline. ^10^ Their lung functions were measured using spirometry. The enrolled cases were regrouped into moderate COPD (50%≤FEV1<80% predicted) and severe COPD (FEV1<50% predicted). The exclusion criteria for COPD participants were: (a) age ≤40 years, (b) patients diagnosed with other respiratory diseases or immunosuppression, and (c) history of antibiotic usage within four weeks prior to sample collection. Healthy controls include individuals ≥40 years of age and those not having any apparent illness. There was no gender-based exclusions or restrictions for recruiting participants. Demographic and clinical information was obtained for the enrolled participants. This study was approved by the Kasturba Medical College and Kasturba Hospital Institutional Ethics Committee (IEC: 479/2019) and the participants were enrolled after providing written informed consent.

### Sample collection, DNA isolation, and 16S rDNA sequencing

The participants were instructed to cough up sputum into a sterile container and samples were transported on ice to the laboratory. All sputum samples were evaluated with routine conventional culturing and an aliquot of it was stored at −80°C for the DNA extraction. The sputum samples were homogenized using an equal volume of 0.2% dithiothreitol (DTT) (Sigma Aldrich, USA), and lysozyme-based Qiagen DNA Mini kit (Qiagen, USA) was used to extract the genomic DNA according to the manufacturer’s protocol. The Qubit 2.0 fluorometer (Thermo Fisher Scientific, USA) was used to measure the purity and concentration of the extracted DNA.

The purified DNA was further processed for the 16S rRNA V3-V4 regions targeted amplification to uncover the bacterial community in sputum samples. Based on the Illumina protocol, ^11^ PCR amplification of V3-V4 hypervariable regions (~456 bp) were performed using the primer pair 341F/785R. Sequencing adapters and dual index barcodes were added with a limited cycle of PCR. After the quality assessment, multiplex amplified libraries were pooled equally and paired-end reads (2X300 bp) were generated using the MiSeq instrument (Illumina, San Diego, CA, United States).

### Bioinformatics analysis

The sequenced raw data were processed using the standard Mothur v1.46.1 pipeline. ^12^ A quality check of the reads was carried out and the low-quality reads and chimeras were removed. Contigs were created from the paired-end reads. Unique sequences were considered by removing the identical sequences. The quality reads were then aligned to the SILVA database and clustered into Operational Taxonomic Units (OTUs) at 97% similarity and taxa level 4, which is similar to the genus for Bacteria. ^13^

### Statistical analysis

The microbial community diversity profiles among the moderate COPD, severe COPD, and healthy groups were analyzed using the alpha and beta-diversity metrics. The Shannon, Simpson, and Chao1 matrices were used to measure bacterial Alpha-diversity and an ANOVA test was done to estimate significant differences. Beta diversity was performed using the Principal Coordinate Analysis (PCoA) method along with the Bray-Curtis index as distance measure and permutational multivariate analysis of variance (PERMANOVA) for the significant measure. The genus biomarkers (discriminative genera among the groups) were identified using linear discriminant analysis effect size (LEfSe) and a cut-off linear discriminant analysis (LDA) score >2.0.

## Results

### Features of the study participants

Sputum samples from COPD cases (12 moderate COPD and 17 severe COPD) and 16 healthy volunteers were included in the study. The participants’ clinical and demographic features like gender, age, BMI, pulmonary function tests (FEV1% predicted, FEV1 and FEV1/FVC values), mMRC dyspnoea score, and whether they were current smokers are summarized in Table 1. At the time of sampling, all COPD patients were in an exacerbated state of the disease and no significant growth of respiratory pathogens was detected in the sputum cultures.

**Table 1.** Clinical and demographic features of the study population.

Features	Moderate COPD (n=12)	Severe COPD (n=17)	Healthy (n=16)
Age, years (mean ± SD)	66 ± 5.8	64 ± 5.8	58 ± 5.3
Gender			
Male (%)	12 (100)	16 (94.1)	10 (62.5)
Female (%)	-	1 (5.9)	6 (37.5)
BMI (kg/m ^2^) (mean ± SD)	25.4 ± 5.6	23.2 ± 5.7	24.2 ± 4.4
Current Smoker (%)	4 (30.8)	2 (11.8)	3 (18.7)
Post-bronchodilator FEV1, % predicted (mean ± SD)	75.1 ± 16.5	30.8 ± 8.6	NA
Post-bronchodilator FEV1, L (mean ± SD)	2.4 ± 0.5	1.8 ± 0.8	NA
Post-bronchodilator FEV1/FVC ratio, % (mean ± SD)	64.7 ± 5.6	61 ± 7.4	NA
mMRC Dyspnoea scale (IQR)	2(2)	3 (3)	NA

### Bacterial diversity in sputum samples

**Figure 1.**
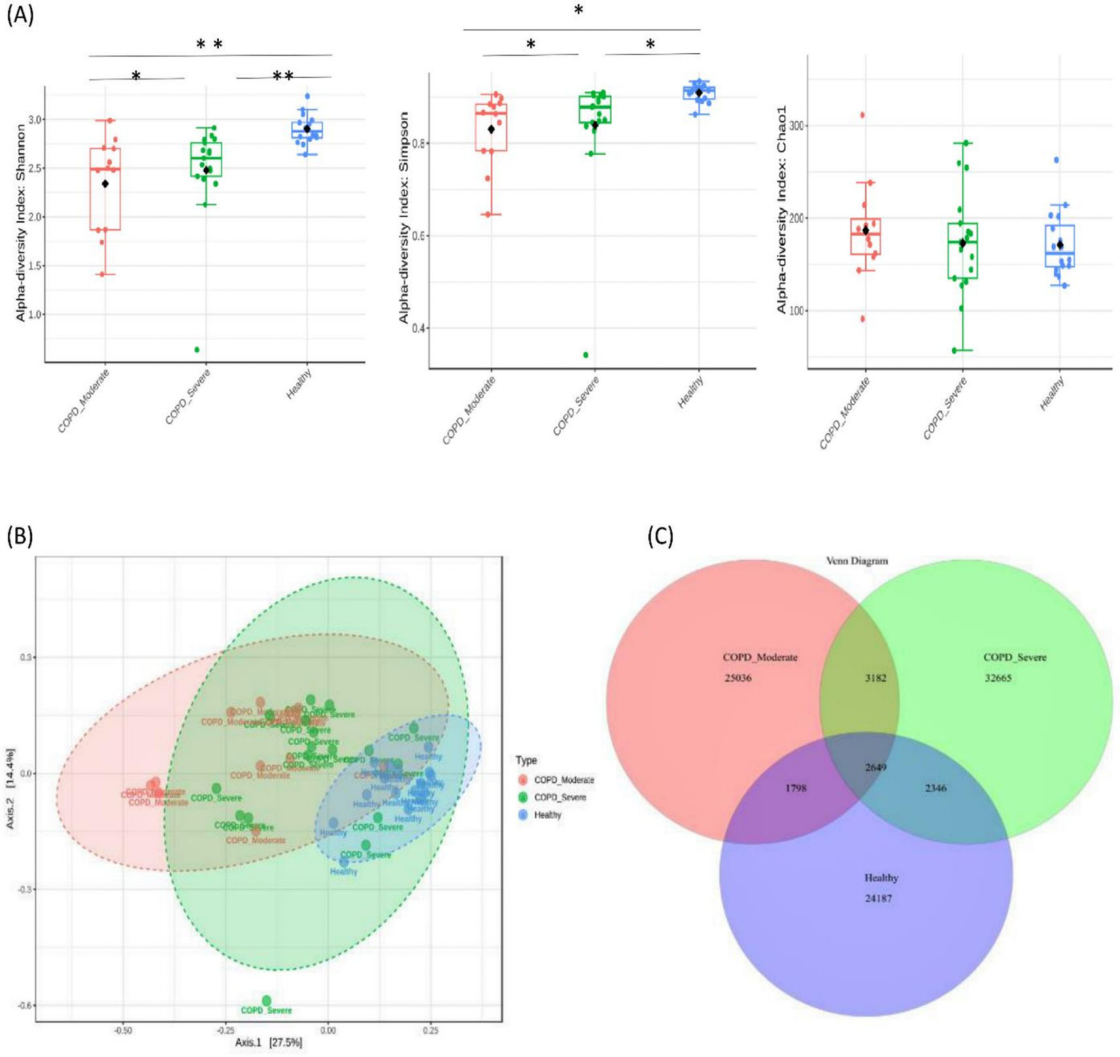
Bacterial diversity in sputum samples of moderate COPD, severe COPD, and healthy groups. (A) The measures of alpha diversity indices showed a significant difference (*p < 0.05 and **p < 0.01, ANOVA test). (B) Principal coordinate analysis (PCoA) revealed a distinct bacterial community clustering among the groups (P< 0.01, PERMANOVA). (C) Venn diagram of the core microbiota in tested samples.

A total of 91,863 OTUs were recovered at a 97% sequence identity with 32,665 OTUs in the patients with moderate COPD, 40,842 OTUs in severe COPD, and 30,980 OTUs in the healthy group. A significantly lower bacterial alpha diversity (Simpson’s and Shannon's index) was observed in moderate COPD and severe COPD samples compared to the healthy group (p<0.05, ANOVA test). The Chao1 index measured the species richness within groups and exhibited no differences (p>0.05) ( Figure 1A). The Beta diversity represented by PCoA showed a significant difference in bacterial community clustering among the groups (p<0.01, PERMANOVA test) ( Figure 1B).

As the Venn diagram of the core sputum microbiota ( Figure 1C) illustrates, out of the total 91863 OTUs 35.6%, 27.2%, and 26.3% OTUs were unique to severe COPD, moderate COPD, and healthy groups respectively. While 2,649 (2.9%) OTUs were shared by all three groups and 5,831 (6.3%) OTUs were shared between moderate and severe COPD groups, 4,447 (4.8%) OTUs were common for moderate COPD and healthy, and, 4,995 (5.4%) OTUs were common for severe COPD and healthy groups.

### The taxonomic profiling of sputum microbiota among groups

**Figure 2.**
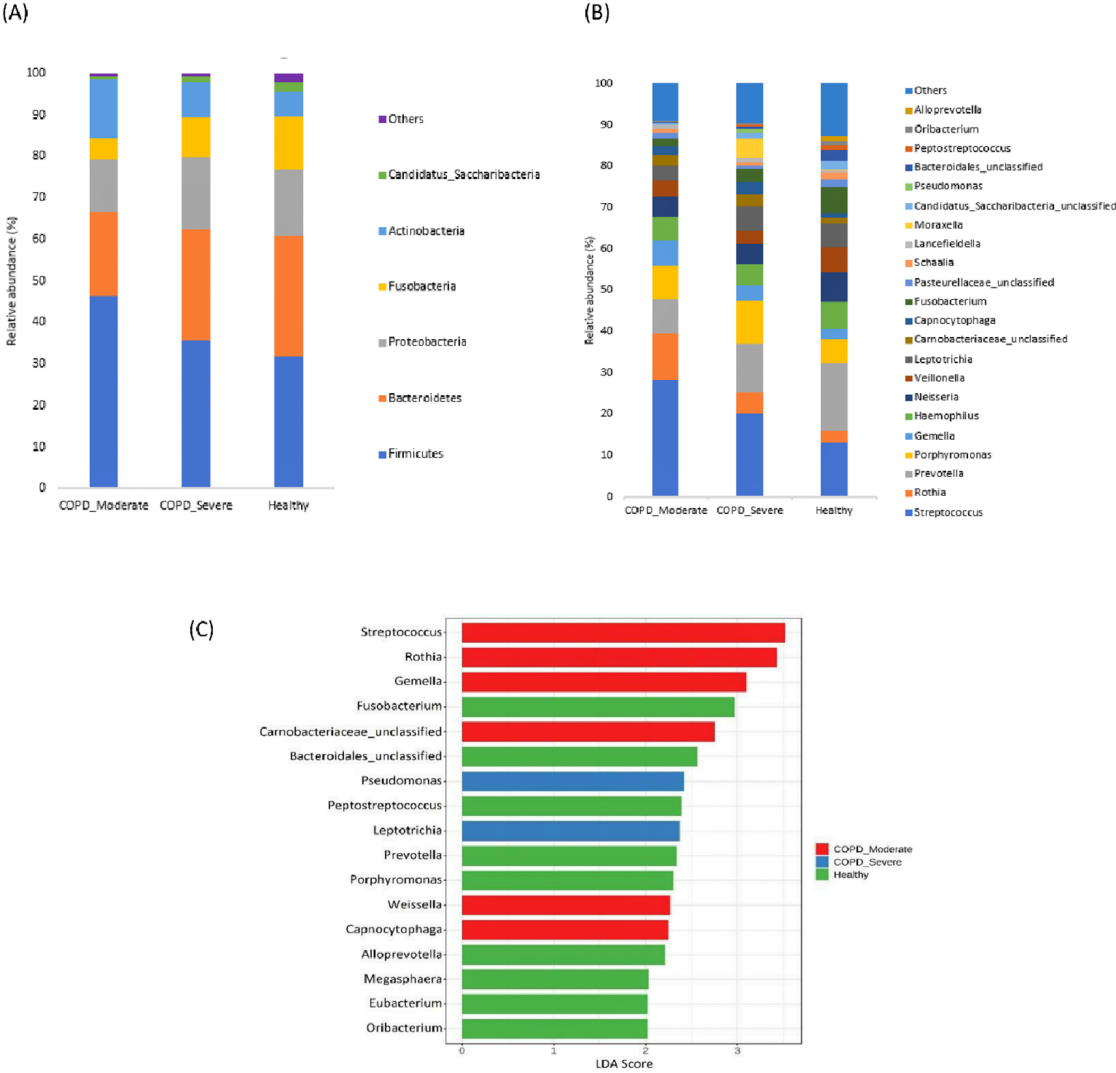
The taxonomic profiling of sputum microbiota among moderate COPD, severe COPD, and healthy groups. (A) Relative abundance at the Phyla level, (B) Relative abundance at the genus level, and (C) LEfSe analysis represent the discriminative genera among the groups (LDA>2).

The most prevalent microbial phyla in the sputum samples of moderate COPD, severe COPD, and healthy were Firmicutes (46.3%, 35.6%, and 31.9%) followed by Bacteroidetes (20.0%, 26.8%, and 28.7%) Proteobacteria (12.9%, 17.4%, and 16.1%), Actinobacteria (14.0%, 8.5%, and 5.9%), and Fusobacteria (5.3%, 9.5%, and 13%) ( Figure 2A). A significantly higher proportion of Firmicutes and Actinobacteria were present in respiratory samples from patients with moderate COPD and Proteobacteria was comparatively increased in severe COPD, whereas in healthy individuals, Bacteroidetes and Fusobacteria were present in a higher abundance compared to both COPD groups.

In the cohort of patients with moderate COPD, the top five most commonly detected genera were *Streptococcus* (28.2%), *Rothia* (11.4%), *Prevotella* (8.3%), *Porphyromonas* (7.9%), and *Gemella* (6.2%). The dominant genera in severe COPD were *Streptococcus* (20.2%), *Prevotella* (11.7%), *Porphyromonas* (10.1%), *Leptotrichia* (5.9%), and *Rothia* (5.3%). In healthy individuals, *Prevotella* (16.5%) was the most prevalent genus, followed by *Streptococcus* (13.0%), *Neisseria* (7.1%), *Fusobacteria* (6.6%), and *Velionella* (6.2%). An increasing abundance of *Streptococcus* (p<0.05), and *Rothia* was observed in moderate COPD samples, whereas *Morexalla* and *Pseudomonas* were relatively higher in severe COPD. Genera like *Prevotella*, and *Fusobacteria* were abundantly present in healthy individuals in comparison to moderate COPD. Figure 2B demonstrates the top bacterial genera present in the sputum of patients with moderate or severe COPD, and healthy groups.

The LEfSe analysis was performed to detect the discriminative genera among the groups ( Figure 2C). In moderate COPD, six marker genera (LDA>2) *Streptococcus, Rothia, Gemella, Carnobacteriaceae, Capnocytophaga,* and *Weissella* were identified. For the severe COPD, genera *Pseudomonas* and *Leptotrichia* were higher, whereas *Fusobacterium, Bacteroidales, Peptostreptococcus, Prevotella, Porphyromonas, Alloprevotella*, etc. were dominant in healthy individuals.

## Discussion

The severity of COPD is often influenced by environmental exposures, host genetic makeup, and airway host-microbiome interactions. This study demonstrated the microbial alpha diversity of moderate and severe COPD groups decreased significantly in comparison to the healthy control group. Our findings added important evidence to the understanding of the microbial population dynamics in COPD. Su *et al.* also reported a decreased bacterial diversity in the acute exacerbations of COPD compared to healthy controls, which was consistent with our findings. ^14^ Ramsheh *et al.* also found a higher Alpha diversity in healthy individuals than in COPD bronchial brush samples. ^15^ The consistent results of these studies indicate the altered microbial diversity in COPD patients compared to healthy and its potential role as a disease marker.

According to our findings, the alpha diversity of moderate COPD declined compared to the severe COPD group. However, Yang *et al.* and Li *et al.* did not observe any significant difference in microbial diversity between mild COPD and severe COPD groups, whereas Garcia-Nuñez *et al.* reported a decreased alpha diversity in advanced COPD compared to moderate-to-severe disease. ^16^^–^^18^ This inconsistency among studies could be due to distinct sampling methods, COPD states and exacerbations, and different geographic regions.

In this study, the most dominant phylum was Firmicutes, predominantly present in all the groups, in concordance with previous reports. ^15^^,^^16^^,^^19^^,^^20^ In healthy controls, we observed a higher proportion of Bacteroidetes and Fusobacteria, likewise reported by Ramsheh *et al.* ^15^ Proteobacteria was present in relatively higher proportions in patients with severe COPD, which is consistent with previous studies examining bronchoalveolar lavage (BAL) ^21^ and sputum ^18^^,^^22^ samples. According to Wang *et al.*, the increased abundance of Proteobacteria might trigger the pro-inflammatory mediators of the host, which leads to dysbiosis of lung microbiomes. ^22^

*Streptococcus* was the predominant genus among the COPD sputum samples, whereas *Prevotella* was significantly higher in healthy controls. A multi-centric study reported that *Prevotella* promotes normal lung function and the severity of COPD increases with its decreasing abundance. ^15^ A lung co-infection mouse model study conducted by Horn KJ *et al.* suggested that an increased abundance of airway *Prevotella* can accelerate the innate immune response and rapid pathogen clearance from the lung. ^23^ In the moderate COPD group, we noted a higher abundance of *Streptococcus* and *Rothia*, which was similar to previously published studies of COPD samples. ^14^^,^^16^^–^^18^^,^^22^^,^^24^ Li W *et al.* also observed a higher abundance of *Rothia* in the mild COPD group compared with the severe group, ^17^ which was negatively correlated with pro-inflammatory markers, which might reduce the disease severity and exacerbation frequency in COPD. ^25^ Another genus, *Morexalla*, which was abundant in severe COPD samples, was likewise reported by Wang *et al.* ^26^ and Ramsheh *et al.* ^15^ According to Wang *et al.*, the relative abundance of *Moraxella* increased during COPD exacerbations and was also linked to the host interferon signaling pathway. ^26^ Ramsheh *et al.* revealed that an increased abundance of *Moraxella* was associated with the expression of the IL-17 and TNF inflammatory pathways, which elicit the severity of COPD. ^15^ These studies indicate that patients might suffer an altered lung microbial diversity during COPD disease severity, which means microbiota is a potential marker to predict the prognosis in COPD cases and may change the disease management.

There are a few limitations of our study. First, the population size of this study was small and the samples were collected from a single center. Second, the virome and mycobiome diversity of sputum were not evaluated. Future multi-centre studies in larger diverse populations are required to conclude the stability and alteration of microbiota in health and disease.

## Conclusions

Our findings suggested a significant loss of the sputum microbiome diversity in patients with COPD. This decrease is more pronounced in patients with severe disease. The dysbiosis of lung microbiota may cause an alteration of the mucosal immune system and further facilitate inflammation in the lung. Therefore, the improved understanding of the link between the respiratory microbiome and disease may offer new opportunities for an alternative management approach for COPD.

## Author contributions

Conceptualization: D.H., K. C.; Methodology: D. H., R. M., K. C.; Data analysis: F. SM., D. H., E. M.; Writing - original draft preparation: D. H.; Writing - review and editing: K. C., V. S., F. SM., N. S.; Supervision: K. C., V. S.

## Data Availability

Zenodo: Sequenced Data COPD and healthy, https://doi.org/10.5281/zenodo.7697770. ^27^ Data are available under the terms of the Creative Commons Attribution 4.0 International license (CC-BY 4.0).
