# Supplementary material for: The altered sputum microbiome profile in patients with moderate and severe COPD exacerbations, compared to the healthy group in the Indian population
Source: F1000Res. 2023 Oct 27;12:528. Originally published 2023 May 22. [Version 4] doi: 10.12688/f1000research.132220.4 (PMC10624950; doi:10.12688/f1000research.132220.4)
Supplement: Peer review history

# Reviewer report: Reviewer response for version 4

**DOI:** 10.5256/f1000research.158079.r218779

**Published:** 2023-11-03

**Authors:** Eduard Monso

**Affiliations:** 1 Airway Inflammation Research Group, Parc Taulí Research and Innovation Institute - I3PT - Parc Taulí Foundation, Barcelona, Spain

The authors have introduced all required changes and specifications. The current version of the manuscript is approved from my side.

Is the work clearly and accurately presented and does it cite the current literature?

Partly

If applicable, is the statistical analysis and its interpretation appropriate?

Partly

Are all the source data underlying the results available to ensure full reproducibility?

Yes

Is the study design appropriate and is the work technically sound?

Partly

Are the conclusions drawn adequately supported by the results?

Yes

Are sufficient details of methods and analysis provided to allow replication by others?

Yes

Reviewer Expertise:

Respiratory diseases / COPD / Lung Cancer / Respiratory microbiome

I confirm that I have read this submission and believe that I have an appropriate level of expertise to confirm that it is of an acceptable scientific standard.

# Reviewer report: Reviewer response for version 3

**DOI:** 10.5256/f1000research.157003.r212696

**Published:** 2023-10-24

**Authors:** Eduard Monso

**Affiliations:** 1 Airway Inflammation Research Group, Parc Taulí Research and Innovation Institute - I3PT - Parc Taulí Foundation, Barcelona, Spain

The authors have addressed satisfactorily the questions raised. The have specified that the samples of COPD patients had been obtained at admission for an exacerbation before the use of any antibiotic. Then, it is clear that they are assessing exacerbation samples, that were compared among them according with the severity pattern, and compared also with samples from healthy subjects. This is important because the title suggests that the authors are studying samples from COPD in stable situation. I think that it would be necessary to specify in the title that the authors are focusing on COPD patients in exacerbation, not in stability, to avoid misunderstandings.

Is the work clearly and accurately presented and does it cite the current literature?

Partly

If applicable, is the statistical analysis and its interpretation appropriate?

Partly

Are all the source data underlying the results available to ensure full reproducibility?

Yes

Is the study design appropriate and is the work technically sound?

Partly

Are the conclusions drawn adequately supported by the results?

Yes

Are sufficient details of methods and analysis provided to allow replication by others?

Yes

Reviewer Expertise:

Respiratory diseases / COPD / Lung Cancer / Respiratory microbiome

I confirm that I have read this submission and believe that I have an appropriate level of expertise to state that I do not consider it to be of an acceptable scientific standard, for reasons outlined above.

# Reviewer report: Reviewer response for version 2

**DOI:** 10.5256/f1000research.153873.r192939

**Published:** 2023-09-01

**Authors:** Eduard Monso

**Affiliations:** 1 Airway Inflammation Research Group, Parc Taulí Research and Innovation Institute - I3PT - Parc Taulí Foundation, Barcelona, Spain

The authors have addressed satisfactorily most of the corrections pointed in the previous review. However, there are a couple of them that still need to be managed.

 1.- (Introduction) The sentence " *Although a high heterogeneity has been observed among the studies, potentially attributed to the underlying health conditions in different populations and the dynamics of microbial ecology in the COPD Indian population, it remains poorly understood*" is misspelled, and needs to be corrected.

 2.- (Results) The sentence " *At the time of sampling, all COPD patients were in an exacerbated state of the disease and no significant growth of respiratory pathogens was detected in the sputum cultures*" specifies that the patients are exacerbated, but the study is restricted to patients that have not used antibiotics recently, suggesting that all them must be stable. This is a main point, because the paper is presented as a stability study. However, is part of the patients were exacerbated the results would be more difficult to be interpreted. This point needs to be clearly specified, and the upper sentence corrected, if misspelled.

Is the work clearly and accurately presented and does it cite the current literature?

Partly

If applicable, is the statistical analysis and its interpretation appropriate?

Partly

Are all the source data underlying the results available to ensure full reproducibility?

Yes

Is the study design appropriate and is the work technically sound?

Partly

Are the conclusions drawn adequately supported by the results?

Yes

Are sufficient details of methods and analysis provided to allow replication by others?

Yes

Reviewer Expertise:

Respiratory diseases / COPD / Lung Cancer / Respiratory microbiome

I confirm that I have read this submission and believe that I have an appropriate level of expertise to state that I do not consider it to be of an acceptable scientific standard, for reasons outlined above.

# Reviewer report: Reviewer response for version 1

**DOI:** 10.5256/f1000research.145128.r182958

**Published:** 2023-07-28

**Authors:** Veronica Ueckermann

**Affiliations:** 1 Department of Internal Medicine, University of Pretoria, Pretoria, Gauteng, South Africa

The manuscript explores loss of microbial diversity in the sputum samples of patients with moderate and severe COPD, and compares the results with a healthy control group. The healthy control group included some smokers and the study population was dominated by males.

 The methodology was well described and both the methods and analysis were appropriate for work in the lung microbiome. The quality of the sputum samples voluntary produced were not discussed and a degree of oropharyngeal contamination is likely.

 Findings are consistent with previously published work and highlight once again signals of dysbiosis in the lung microbiome of patients with COPD. Had the sample size allowed, a comparison between current and previous smokers may have provided additional insights.  

 The limitations of the study were acknowledged - both the small sample size and the fact that the virome (which is becoming increasingly important in COPD) was not evaluated.  

 Although the findings of the study were not completely novel, the differences by disease severity was interesting and the study does add to a growing body of literature that enhances the understanding of the lung microbiome in respiratory disease. The methodology was sound and the discussion well written.

Is the work clearly and accurately presented and does it cite the current literature?

Yes

If applicable, is the statistical analysis and its interpretation appropriate?

Yes

Are all the source data underlying the results available to ensure full reproducibility?

Yes

Is the study design appropriate and is the work technically sound?

Yes

Are the conclusions drawn adequately supported by the results?

No

Are sufficient details of methods and analysis provided to allow replication by others?

Yes

Reviewer Expertise:

Infectious diseases, Critical care, lung microbime, Tuberculosis

I confirm that I have read this submission and believe that I have an appropriate level of expertise to confirm that it is of an acceptable scientific standard.

# Reviewer report: Reviewer response for version 1

**DOI:** 10.5256/f1000research.145128.r175709

**Published:** 2023-07-20

**Authors:** Eduard Monso

**Affiliations:** 1 Airway Inflammation Research Group, Parc Taulí Research and Innovation Institute - I3PT - Parc Taulí Foundation, Barcelona, Spain

The authors described the respiratory microbiome in healthy subjects and COPD patients with different levels of severity, confirming with their study the characteristics of the COPD microbiome previously reported in other communities. The main value of the paper, accordingly, is the analysis of a community living in a specific geographic area, not previously focused. There are some concerns that need to be raised, however, as followed.

1. The title state that the study focus on the lung microbiome dynamics. However, considering that the studied sample is the sputum, it can only be representative of the bronchial microbiome, not the lung. Furthermore, because there has been a single sampling in the studied population, the study cannot be considered "dynamic".
2. In the description of the features of the study participants, it is said that they are exacerbated. I think that this is a typewriting mistake, because all the text suggests that the participants are stable (they have not used antibiotics the previous months).
3. In the table, moderate COPD patients have an average FEV1/FVC of 90. If that is the case, most of them do not suffer from COPD, and probably have only chronic bronchitis. This is a main point that needs to be clarified.
4. The comparisons would have been much more clear if all COPD patients are compared with healthy subjects, and later, excluding healthy subjects, severe and moderate COPD patients were compared. 

Is the work clearly and accurately presented and does it cite the current literature?

Partly

If applicable, is the statistical analysis and its interpretation appropriate?

Partly

Are all the source data underlying the results available to ensure full reproducibility?

Yes

Is the study design appropriate and is the work technically sound?

Partly

Are the conclusions drawn adequately supported by the results?

Yes

Are sufficient details of methods and analysis provided to allow replication by others?

Yes

Reviewer Expertise:

Respiratory diseases / COPD / Lung Cancer / Respiratory microbiome

I confirm that I have read this submission and believe that I have an appropriate level of expertise to state that I do not consider it to be of an acceptable scientific standard, for reasons outlined above.

# Response

**Published:** 2023-07-26

**Authors:** Kiran Chawla

**Affiliations:** Microbiologt, Manipal Academy of Higher Education, Manipal, Karnataka, India

**Response to the Reviewer Comments:**

**Query 1.** The title state that the study focus on the lung microbiome dynamics. However, considering that the studied sample is the sputum, it can only be representative of the bronchial microbiome, not the lung. Furthermore, because there has been a single sampling in the studied population, the study cannot be considered "dynamic".

**Response:** Thank you for your valuable suggestions. In this study, we characterized sputum, an easily obtainable non-invasive sampling method that represents a mix of upper and lower respiratory tracts, used for routine diagnosis of lower-respiratory tract infection.

 The term dynamics have been used here to refer to the changes in microbiome composition among the groups

**Query 2.** In the description of the features of the study participants, it is said that they are exacerbated. I think that this is a typewriting mistake, because all the text suggests that the participants are stable (they have not used antibiotics the previous months).

**Response:** The enrolled participants were the known cases of COPD, admitted to our hospital for the management of the latest exacerbation state, and a sample was collected before administrating antibiotics. So the patients have not used antibiotics in the previous months. Also, one of our exclusion criteria is antibiotic usage within four weeks prior to sample collection since antibiotics can alter the microbiome compositions. 

**Query 3.** In the table, moderate COPD patients have an average FEV1/FVC of 90. If that is the case, most of them do not suffer from COPD, and probably have only chronic bronchitis. This is a main point that needs to be clarified.

**Response: **Thank you, Sir, for mentioning this point. This was a typewriting error, which we didn’t notice earlier. We rechecked our data and there was an error in that particular row of that table. It should be the moderate COPD average FEV1/FVC was 64.7 ± 5.6 and Severe COPD 61 ± 7.4

**Query 4. **The comparisons would have been much more clear if all COPD patients are compared with healthy subjects, and later, excluding healthy subjects, severe and moderate COPD patients were compared.

**Response:** We compared the sputum microbiome in healthy, severe, and moderate COPD patients as per the objective. We admire your valuable suggestions and we will consider them for our future studies.
